# Effectiveness of physical activity and sedentary behaviour interventions delivered following cancer screening or suspected cancer referral: a systematic review and meta-analysis protocol

**DOI:** 10.1136/bmjopen-2026-127499

**Published:** 2026-10-29

**Authors:** Dharani Yerrakalva, Ben Gregory

**Affiliations:** 1 University of Cambridge, Cambridge, UK; 2 School of Clinical Medicine, University of Cambridge, Cambridge, UK

**Keywords:** Exercise, Primary Prevention, Randomized Controlled Trial

## Abstract

**Abstract:**

**Introduction:**

Higher levels of physical activity (PA) and lower levels of sedentary behaviour are associated with reduced cancer incidence and premature mortality. However, interventions to increase PA and reduce sedentary time often achieve modest and short-term behaviour change. Significant health events may represent ‘teachable moments’ when individuals are particularly receptive to behaviour change. Cancer screening and referral for investigation of suspected cancer may provide such opportunities particularly following a negative screening result or non-cancer diagnosis. However, the effectiveness of PA and sedentary interventions delivered during these clinical pathways has not been systematically evaluated.

**Methods and analysis:**

This systematic review and meta-analysis protocol has been developed in accordance with Preferred Reporting Items for Systematic Review and Meta-Analysis Protocols (PRISMA-P) guidance. MEDLINE (Ovid), Embase (Ovid), Web of Science Core Collection and the Cochrane Central Register of Controlled Trials will be searched from January 2008 onwards, supplemented by searches of relevant trial registries and reference lists. Eligible studies will include randomised and cluster-randomised controlled trials involving adults (≥18 years) who have undergone cancer screening or have been referred for investigation of suspected cancer and subsequently received a non-cancer diagnosis. Interventions must explicitly aim to increase PA and/or reduce sedentary behaviour and be delivered at or following cancer screening or following referral for suspected cancer and receipt of a non-cancer diagnosis. Primary outcomes will be change in objectively measured PA and change in objectively measured sedentary time. Two reviewers will independently screen studies, extract data and assess risk of bias using the appropriate Risk of Bias 2 tool for individually randomised or cluster-randomised trials. Intervention content will be coded using the Behaviour Change Technique Taxonomy. Where appropriate, random-effects meta-analysis will be conducted. Secondary objectives will examine intervention functions and behaviour change techniques in relation to the magnitude and direction of effects and compare mobile health (mHealth) with non-mHealth interventions. Certainty will be assessed using the Grading of Recommendations Assessment, Development and Evaluation approach (GRADE).

**Ethics & dissemination:**

Ethics review was not required as this is a systematic review protocol. Findings will be disseminated through peer-reviewed publication, conference presentations and engagement with relevant cancer prevention and public health stakeholders.

**PROSPERO registration number:**

CRD420261481574.

**STRENGTHS AND LIMITATIONS OF THIS STUDY**

- This review examines the importance of **when** physical activity interventions are delivered, rather than simply whether they are effective, testing the hypothesis that cancer screening and investigation of suspected cancer may represent ‘teachable moments’ for behaviour change.
- The review distinguishes between two clinically different opportunities for intervention: **cancer screening** and **investigation of suspected cancer followed by a non-cancer diagnosis**, allowing potential differences in the behavioural response to these contexts to be explored.
- By coding intervention functions and behaviour change techniques, the review will examine not only whether interventions are effective, but **which intervention components may be particularly suited to delivery within these cancer-related clinical pathways**.
- The review will specifically examine whether **mobile health interventions**, which may be readily delivered at scale alongside routine screening and diagnostic pathways, differ in effectiveness from non-digital approaches.
- The number and heterogeneity of studies addressing these relatively specific clinical time points may be limited, potentially restricting statistical power for meta-analysis and making it difficult to distinguish effects of intervention content from effects of the clinical context in which interventions are delivered.

## Introduction

 Insufficient physical activity (PA) and prolonged sedentary behaviour are major determinants of population health. Higher levels of PA are associated with lower risks of several cancers, including colorectal, breast and endometrial cancer, with evidence also supporting associations with other common cancers.^1 2^ Prospective evidence further suggests that changes in PA over time are associated with subsequent cancer and premature mortality outcomes.^3^ Increasing PA is therefore an important component of cancer prevention and broader strategies to improve population health. Despite this evidence, achieving sustained increases in PA at population level remains challenging. Numerous behavioural interventions have demonstrated short-term improvements in PA, but effects frequently attenuate over longer follow-up.^4 5^ Interventions delivered through healthcare settings may provide an opportunity to reach large numbers of adults and can be effective in increasing PA, but sustained behavioural change remains difficult to achieve. Consequently, identifying circumstances in which people may be particularly receptive to PA interventions is an important priority.

One potential opportunity is provided by significant health-related events. The concept of a ‘teachable moment’ proposes that an event that prompts individuals to reconsider their health or mortality may temporarily increase motivation and openness to behaviour change. Delivering behavioural interventions at these points could therefore potentially increase their effectiveness compared with delivering the same intervention at an unrelated time.^6^ Cancer screening may represent one such opportunity: it provides contact with healthcare services, can increase awareness of personal cancer risk and may prompt individuals to consider the relationship between their health behaviours and future disease. A review of interventions delivered within cancer screening programmes found that screening may provide a valuable setting for lifestyle prevention, although evidence for effects on physical activity was inconsistent.^7^ For individuals who subsequently receive a negative screening result or a non-cancer diagnosis, reassurance may also provide an opportunity to shift attention towards prevention and longer-term health.

There are therefore plausible reasons why the timing of a PA or sedentary behaviour intervention around these clinical pathways might influence its effectiveness. However, the experience of screening or referral may not uniformly increase readiness for behaviour change. For some individuals, these events may instead generate anxiety, reassurance without behavioural change or a focus on other health concerns. Furthermore, the optimal timing of an intervention is uncertain: an intervention could be delivered during screening or investigation, immediately following a result or subsequently as part of routine follow-up. Understanding whether these contexts represent effective opportunities for PA promotion therefore requires evaluation of the available intervention evidence.

The nature of interventions delivered in these settings may also be important. Mobile health (mHealth) technologies, including smartphone applications and wearable devices, offer potentially scalable approaches that can provide personalised advice, feedback and prompts with relatively limited demands on healthcare professionals. They may be particularly suitable for delivery alongside screening and diagnostic pathways, where opportunities for face-to-face behavioural support may be limited and not scalable. However, it is unclear whether digital approaches are more effective than non-digital interventions when delivered in these specific contexts.

Beyond determining whether interventions increase PA and reduce sedentary time, understanding their behavioural content may help identify approaches that are transferable to future interventions. Behavioural interventions can incorporate multiple functions and behaviour change techniques, such as education, goal setting, feedback, social support and action planning. Systematically characterising these components and mapping them against the direction and magnitude of intervention effects may help identify intervention features that warrant further investigation in future interventions targeted to cancer prevention pathways. The Behaviour Change Wheel and Behaviour Change Technique Taxonomy V.1 provide established frameworks for this purpose.^8 9^

To date, no systematic review has specifically examined the effectiveness of PA and sedentary behaviour interventions delivered at or following cancer screening, or following referral for suspected cancer resulting in a non-cancer diagnosis. A synthesis of this evidence is therefore needed to determine whether these clinical pathways represent promising opportunities for PA promotion, and to explore how intervention characteristics may influence effectiveness.

Although cancer screening and investigation of suspected cancer may provide opportunities for behaviour change, this review will not be able to determine whether these clinical pathways represent more effective ‘teachable moments’ than other points in the healthcare journey unless eligible trials directly compare intervention delivery at different time points or clinical contexts. Rather, the review will establish whether physical activity and sedentary behaviour interventions delivered within these pathways are effective compared with their respective control conditions. Any conclusions regarding the potential importance of intervention timing will therefore be considered hypothesis-generating, unless supported by direct comparative evidence.

## Objectives

The primary objective of this systematic review is to determine the effectiveness of PA and sedentary behaviour interventions delivered at or following cancer screening, or following referral for suspected cancer resulting in a non-cancer diagnosis, in increasing PA and reducing sedentary time among adults.

The secondary objectives are to:

1. Characterise intervention functions and examine them descriptively in relation to the direction and magnitude of intervention effects.
2. Characterise behaviour change techniques and examine them descriptively in relation to the direction and magnitude of intervention effects.
3. Compare the effectiveness of mHealth and non-mHealth interventions.

## Methods

This systematic review and meta-analysis protocol has been developed in accordance with the Preferred Reporting Items for Systematic Review and Meta-Analysis Protocols (PRISMA-P) guidance.^10^ The protocol has been registered prospectively with PROSPERO (CRD420261481574).

### Eligibility criteria

Eligibility criteria will be defined a priori according to population, intervention, comparator, outcomes and study design.

#### Population

Studies will be eligible if they include adults aged 18 years or older who have either:

1. undergone cancer screening; or
2. been referred for investigation of suspected cancer and subsequently received a non-cancer diagnosis.

Studies involving individuals with a confirmed cancer diagnosis will be excluded. Where studies include a mixed population, the study will be eligible if data for participants meeting the review population criteria can be separately identified.

### Intervention

Eligible interventions will be those with the explicit aim of increasing physical activity and/or reducing sedentary behaviour. Interventions must be delivered at the time of cancer screening, following cancer screening, or following referral for suspected cancer and receipt of a non-cancer diagnosis.

### Comparator

Eligible comparator conditions will include usual care, no intervention, wait-list control, minimal intervention or a lower-intensity intervention.

### Outcomes

The primary outcomes will be: (1) change in objectively measured physical activity and (2) change in objectively measured sedentary time. Eligible objective measures of physical activity will include pedometer-measured steps per day, accelerometer- or device-derived moderate-to-vigorous physical activity and other device-derived physical activity metrics. Eligible sedentary behaviour measures will include accelerometer- or device-derived sedentary time. Objective measurement is required for a study to be included in the review; eligible studies reporting only self-reported physical activity or sedentary behaviour outcomes will be excluded. Where a study reports multiple objective physical activity metrics, conceptually distinct outcomes (eg, steps/day, MVPA and total physical activity) will be synthesised separately rather than selected on the basis of statistical significance. Where multiple follow-up assessments are reported, the assessment closest to the end of the intervention will be prioritised for the primary analysis; longer-term follow-up assessments will be synthesised separately where sufficient data are available.

### Study design

Randomised controlled trials and cluster-randomised controlled trials will be eligible for inclusion. Studies published before January 2008 will be excluded to focus the review on contemporary cancer-screening and diagnostic pathways and contemporary physical activity interventions, including the period in which device-based and mobile-health approaches became increasingly relevant to intervention delivery and outcome assessment. The review will be restricted to English-language publications because resources for translation are not available. The potential for date and language restrictions to introduce selection bias will be acknowledged when interpreting the findings.

### Information sources

Four electronic databases will be searched: MEDLINE via Ovid, Embase via Ovid, Web of Science Core Collection and Cochrane Central Register of Controlled Trials (CENTRAL). ClinicalTrials.gov and the WHO International Clinical Trials Registry Platform will also be searched to identify ongoing or unpublished eligible trials. Reference lists of all included studies and relevant systematic reviews and commentaries will be screened to identify additional eligible studies. The electronic database and trial-registry searches will be rerun before the final analysis to identify newly published, registered or completed studies.

### Search strategy

The search strategy will combine terms relating to three key concepts: physical activity and sedentary behaviour; cancer screening or investigation of suspected cancer; and trials or interventions designed to promote physical activity and/or reduce sedentary behaviour (online supplemental file 1). The strategy will use controlled vocabulary and free-text terms, with truncation and proximity operators where appropriate. Cancer-related terminology will include general screening and suspected-cancer terms alongside terminology for specific screening procedures and referral pathways. The strategy will be adapted for the syntax and indexing terms of each database. The strategy will be reviewed by an experienced information specialist using the PRESS framework, where feasible, before the final search is undertaken. The search will be repeated prior to final analysis.

### Study selection

Search results will be imported into Covidence, where duplicate records will be identified and removed. Two reviewers will independently screen titles and abstracts against the predefined eligibility criteria. Records considered potentially eligible by either reviewer, or for which eligibility cannot be determined from the title and abstract, will proceed to full-text assessment. Two reviewers will independently assess the full text of potentially eligible reports. Reasons for exclusion at the full-text stage will be recorded. Disagreements at either stage will initially be resolved through discussion between the two reviewers; where consensus cannot be reached, a third reviewer will adjudicate. The study selection process will be reported using a Preferred Reporting Items for Systematic Reviews and Meta-Analyses (PRISMA) flow diagram.^10^

### Data extraction

Two reviewers will independently extract data using a standardised data extraction form developed for the review. Extracted information will include study characteristics (design, setting, country, recruitment method and funding source), participant characteristics (sample size and demographic characteristics), cancer screening or suspected-cancer referral context, timing of the intervention in relation to screening or referral and diagnostic outcome, intervention characteristics (content, duration, intensity, delivery mode and use of digital technologies), comparator characteristics, physical activity and sedentary behaviour outcome measures, baseline and follow-up assessment time points, intervention adherence and participant retention and information required for assessment of risk of bias. Where multiple publications relate to the same study, reports will be linked and data will be extracted at the study level to avoid double counting participants. Where information required to determine eligibility, risk of bias or quantitative synthesis is missing or unclear, study investigators will be contacted for clarification or additional data. Where data remain unavailable, analyses will use the information reported in the publication and the implications of missing information will be considered in the risk-of-bias assessment and interpretation of findings.

### Coding of intervention content

Intervention content will be characterised using established behavioural science frameworks. Two trained reviewers will independently code intervention functions using the Behaviour Change Wheel^8^ and behaviour change techniques using the Behaviour Change Technique Taxonomy version 1 (BCTTv1).^9^ Coding will be based on information available in the primary publication and, where necessary, associated protocols, online supplemental materials or other linked publications. Intervention functions and behaviour change techniques will be mapped descriptively against the direction and magnitude of intervention effects. Where sufficient studies are available, exploratory comparisons may be undertaken; these will be interpreted as associations and not as evidence that individual intervention components causally determine effectiveness. Disagreements in coding will be resolved through discussion and, where necessary, consultation with a third reviewer.

### Risk of bias assessment

Risk of bias will be assessed independently by two reviewers for each relevant outcome/result. Individually randomised controlled trials will be assessed using the standard Cochrane risk of bias 2 (RoB 2) tool,^11^ while cluster-randomised controlled trials will be assessed using the RoB 2 tool and guidance specific to cluster-randomised trials. Risk-of-bias judgements will therefore relate to particular results rather than a single generic study-quality classification. Disagreements will be resolved through discussion and, where necessary, consultation with a third reviewer. Risk-of-bias assessments will inform interpretation of the findings and planned sensitivity analyses.

### Data synthesis

#### Narrative synthesis

A narrative synthesis will be undertaken for all included studies. Studies will be summarised according to population and clinical context, intervention characteristics, delivery mode, timing of intervention, intervention functions, behaviour change techniques, comparator, outcome measures, follow-up duration, adherence and risk of bias. Particular attention will be given to differences between interventions delivered around cancer screening and those delivered following investigation of suspected cancer resulting in a non-cancer diagnosis. The synthesis will also describe whether interventions were delivered digitally or through non-digital approaches and whether intervention content differed according to the clinical context.

#### Quantitative synthesis

Given anticipated clinical and methodological heterogeneity, random-effects meta-analysis will be undertaken where at least two studies are sufficiently comparable. Between-study variance will be estimated using restricted maximum likelihood. For meta-analyses including three or more studies, Hartung-Knapp adjustments will be used to calculate CIs around pooled effects. Where the Hartung-Knapp variance estimate produces a narrower CI than the conventional random-effects method, a modified Hartung-Knapp approach with a variance correction will be applied. The chosen adjustment and any deviations will be documented in the final review. Where only two studies are available, conventional random-effects CIs will be presented, and the substantial uncertainty in estimating between-study heterogeneity will be acknowledged. Analyses using alternative CI methods will be considered as sensitivity analyses where appropriate. For continuous outcomes measured using the same metric, pooled mean differences (MDs) with 95% CIs will be calculated. Where studies assess the same underlying construct using different measurement scales or metrics that can appropriately be combined, standardised mean differences (SMDs) with 95% CIs will be calculated.

Physical activity and sedentary behaviour will be synthesised separately. Where both change-from-baseline and endpoint data are reported, change-from-baseline data will be used preferentially; endpoint data will be used where change data are unavailable. Where SD are not reported, they will be derived from other available statistics using established Cochrane methods where possible; study investigators will be contacted where required. If an SD cannot be obtained or derived, the study will not contribute to the relevant pooled estimate. Effect directions will be harmonised so that pooled estimates have a consistent interpretation. Where multiple follow-up assessments are available, the assessment closest to the end of the intervention will be used for the primary analysis, with longer-term follow-up synthesised separately where sufficient data are available. For cluster-randomised trials, appropriately cluster-adjusted effect estimates and standard errors will be used where reported. Where clustering has not been accounted for, the effective sample size will be adjusted using the reported intracluster correlation coefficient (ICC), or an appropriate ICC from a comparable study where justified; assumptions will be reported and explored in sensitivity analysis where possible.

#### Assessment of heterogeneity

Clinical and methodological heterogeneity will be assessed by examining differences in study populations, cancer-related clinical context, intervention characteristics, comparators, outcome measurement and follow-up duration. Statistical heterogeneity will be assessed using Cochran’s Q (χ²) test and the I² statistic. An I² value above 50% will be considered indicative of potentially substantial statistical heterogeneity, although interpretation will take account of the number of studies and the magnitude and direction of effects.

### Subgroup analyses

Where sufficient data are available, prespecified exploratory subgroup analyses will examine: (i) mHealth versus non-mHealth interventions; (ii) interventions targeting physical activity alone versus those targeting both physical activity and sedentary behaviour; and (iii) cancer-screening populations versus populations referred for investigation of suspected cancer. Mhealth will be defined as ‘medical and public health practice supported by mobile devices, such as mobile phones, patient monitoring devices, personal digital assistants, and other wireless devices’.^12^

Where at least ten studies contribute to a meta-analysis and sufficient studies are represented within each subgroup, differences between subgroups will be examined using a formal test for subgroup interaction, or random-effects meta-regression where appropriate. The number of studies, distribution of relevant study characteristics and potential confounding will be considered when determining whether formal comparisons are appropriate. Where data are insufficient, subgroup findings will be presented descriptively.

Comparisons between mHealth and non-mHealth interventions will be regarded as exploratory indirect comparisons between different sets of trials, unless eligible studies directly randomise participants between these intervention types. Differences in pooled effects will not be interpreted as evidence of comparative superiority without considering differences in populations, clinical contexts, intervention intensity, comparators and other potential confounders.

Intervention functions (using the Behaviour Change Wheel^8^) and BCTs (using the Behaviour Change Technique Taxonomy version 1^9^) will be mapped descriptively against the direction and magnitude of effect estimates. Any exploratory component-effect comparisons will be interpreted cautiously and will not be considered evidence of causal effects of individual intervention components.

#### Sensitivity analyses

Sensitivity analyses will be undertaken to examine the robustness of findings to risk of bias. Where sufficient studies are available, analyses will be repeated excluding studies judged to have an overall high risk of bias for the outcome/result under consideration. Where cluster-randomised trials require assumptions about an ICC, sensitivity analyses using plausible alternative ICC values will be undertaken where feasible.

#### Assessment of publication bias

Where at least 10 studies contribute to a meta-analysis, potential small-study effects and publication bias will be explored using funnel plots and Egger’s test.

#### Certainty of evidence

The certainty of evidence for the primary outcomes will be assessed using the GRADE approach.^13^ Evidence will be considered across relevant domains, including risk of bias, inconsistency, indirectness, imprecision and publication bias. The certainty of evidence will be reported alongside the main findings to support interpretation of the clinical implications of the review.

#### Review status and anticipated completion

At the time of protocol revision (October 2026), the review is at the pre-literature search phase. We anticipate completing study selection and data extraction by December 2026, with data synthesis and manuscript preparation completed by April 2027. Any amendments to the registered protocol will be documented and reported in the final review.

#### Ethics & dissemination

Ethics review was not required as this is a systematic review protocol. Findings will be disseminated through publication in a peer-reviewed journal, presentation at relevant scientific conferences and engagement with stakeholders involved in cancer prevention, screening and public health. Where appropriate, findings will be communicated in accessible formats to relevant patient and public audiences.

## Supplementary material

10.1136/bmjopen-2026-127499 online supplemental file 1

## Data Availability

No data are available.
