# Supplementary material for: Effectiveness of physical activity and sedentary behaviour interventions delivered following cancer screening or suspected cancer referral: a systematic review and meta-analysis protocol
Source: BMJ Open. 2026 Oct 29;16(10):e127499. doi: 10.1136/bmjopen-2026-127499 (PMC13723863; doi:10.1136/bmjopen-2026-127499)
Supplement: Supplementary file 1 [file bmjopen-2026-127499.reviewer_comments.pdf]

PEER REVIEW HISTORY

BMJ Open publishes all reviews undertaken for accepted manuscripts. Reviewers are asked to complete a checklist review form and are provided with free text boxes to elaborate on their assessment. These free text comments are reproduced below.

ARTICLE DETAILS

Title (Provisional)

Effectiveness of physical activity and sedentary behaviour interventions delivered following cancer screening or suspected cancer referral: a systematic review and meta-analysis protocol

Authors

Yerrakalva, Dharani; Gregory, Ben

VERSION 1 - REVIEW

Reviewer 1

Name Ciaccioni, Simone

Affiliation University of Rome 'Foro Italico' Faculty of Sport and Exercise Sciences, Department of Movement, Human and Health Sciences, Division of Human Movement and Sport Sciences

Date 28-Sep-2026

COI None

This protocol addresses a relevant and clearly focused question concerning the effectiveness of physical activity and sedentary behaviour interventions delivered at potentially important "teachable moments" associated with cancer screening and investigation of suspected cancer. The distinction between screening populations and people receiving a non-cancer diagnosis after suspected-cancer referral is particularly useful. The prospective PROSPERO registration, duplicate screening and extraction, planned RoB 2 assessment, intervention-content coding, meta-analysis where appropriate, and GRADE assessment are strengths. However, several methodological details should be clarified prospectively.

1. The proposed MEDLINE strategy appears relatively restrictive for the breadth of the research question. In particular, the first block contains only "intervention", "randomised control trial", "advice" and Telemedicine[Mesh], while the screening/referral block may not retrieve studies indexed primarily through specific screening procedures or pathways. Consider broadening the terminology to include relevant trial terms and specific cancer-screening or referral terminology, and ensure that mHealth/digital interventions are adequately represented if they constitute a prespecified subgroup. The authors should also

consider trial registries and other grey-literature sources, particularly because publication bias is explicitly planned for assessment. The restrictions to English-language studies and publications from January 2008 onwards should be explicitly justified, as both restrictions could introduce selection bias. Ideally, the final strategy should be reviewed by an information specialist or using a structured peer-review approach such as PRESS.

2. Outcome eligibility and prioritisation require further specification. It is currently unclear whether objectively measured physical activity or sedentary behaviour is an eligibility requirement, or whether studies using only self-reported outcomes could be included but would not contribute to the primary outcome. Please state this explicitly. Furthermore, studies may report steps/day, MVPA, total activity, sedentary time and several device-derived metrics at multiple follow-up periods. A prespecified hierarchy for selecting outcomes and time points would reduce the possibility of selective inclusion of results. Physical activity and sedentary time should also be treated clearly as distinct outcomes rather than implicitly combined simply because both are device-derived.

3. The quantitative synthesis plan needs additional detail. The manuscript specifies random-effects meta-analysis, MD/SMD and preference for change scores, but states that the approach for combining different forms of outcome data "will be specified before analysis". Such decisions should preferably be specified in the protocol itself. Please clarify the random-effects estimator, handling of change versus endpoint scores, missing SDs, direction of scales, and selection of follow-up periods. Since cluster-randomised trials are eligible, the protocol should also describe how clustering will be handled when trial estimates have not already been appropriately adjusted. The expression "OP test" for heterogeneity should also be checked; if Cochran's Q test is intended, this should be corrected.

4. Subgroup/component analyses need clearer operational definitions. The proposal to compare "BCTs and intervention functions utilised in effective vs non-effective interventions" requires a priori definition of what constitutes an "effective" intervention. Dichotomising trials according to statistical significance could be misleading, particularly with small studies and heterogeneous outcomes. If the number of studies is limited, descriptive mapping of BCTs/intervention functions against effect estimates may be more defensible than inferring that individual components are associated with effectiveness. Please specify the intended analytic approach and avoid causal interpretation of component-effect associations.

5. Risk-of-bias assessment should distinguish individually randomised and cluster-randomised trials. RoB 2 provides specific guidance/tools for cluster-randomised trials, and risk-of-bias judgements are generally made for particular results rather than assigning a single generic "study quality" classification. Please specify the relevant RoB 2 approach and align the planned sensitivity analyses accordingly.

6. PRISMA-P reporting and methodological references should be corrected. The manuscript states that the protocol was developed according to PRISMA-P but reference 8 is the 2009 PRISMA Statement rather than the PRISMA-P 2015 statement. Please cite the appropriate

PRISMA-P publication and cross-check the submitted PRISMA-P checklist against the manuscript. For example, the checklist indicates complete reporting of data-collection processes, although procedures for contacting investigators or handling missing/uncertain information are not clearly described. The manuscript should also state the current review status and anticipated dates/timeline, as appropriate for a protocol.

7. Please align the Abstract and main Methods more closely. The abstract describes adherence as a secondary outcome, whereas the formal secondary objectives focus on intervention functions, BCTs and mHealth versus non-mHealth interventions. Please ensure that primary and secondary outcomes/objectives are defined consistently throughout the abstract, eligibility criteria and synthesis plan.

8. A recent methodological protocol may strengthen the Methods rationale. The authors could consider referencing Ciaccioni et al., MSCA HOPE - Physical education in hospital schools: A protocol paper for systematic and umbrella reviews with policy framework analysis (Open Research Europe, 2026; doi:10.12688/openreseurope.23993.2). Its relevance is methodological: it provides a recent example in the physical-activity field of prospective, preregistered evidence synthesis combining explicitly defined eligibility criteria and structured appraisal of methodological quality and evidence certainty. The most appropriate location would therefore be in the Methods, immediately after the paragraph describing protocol development/registration, or when explaining the rationale for prospectively defining appraisal and synthesis procedures. A possible formulation is: "Recent protocols for evidence synthesis in the broader physical activity field have similarly emphasised prospective specification of complementary review methods, methodological appraisal and assessment of evidence certainty (Ciaccioni et al., 2026)."

Finally, the manuscript is generally clearly written. Minor copy-editing is recommended for typographical and formatting inconsistencies, but these do not materially affect comprehensibility.

VERSION 1 - AUTHOR RESPONSE

Response to Reviewer 1

Effectiveness of physical activity and sedentary behaviour interventions delivered following cancer screening or suspected cancer referral: a systematic review and meta-analysis protocol

We thank the reviewer for the thoughtful and constructive assessment. We have revised the protocol to clarify the search strategy, outcome eligibility and prioritisation, synthesis methods, intervention-component analyses, risk-of-bias assessment, reporting and consistency between the abstract and Methods. Amendments are highlighted in the accompanying marked-up manuscript. Reviewers comments are in blue below, responses are in black and changes are highlighted in italics.

Comment 1. Search strategy and eligibility restrictions: The proposed MEDLINE strategy appears relatively restrictive for the breadth of the research question. In particular, the first block contains only "intervention", "randomised control trial", "advice" and Telemedicine[Mesh], while the screening/referral block may not retrieve studies indexed primarily through specific screening procedures or pathways. Consider broadening the terminology to include relevant trial terms and specific cancer-screening or referral terminology, and ensure that mHealth/digital interventions are adequately represented if they constitute a prespecified subgroup. The authors should also consider trial registries and other grey-literature sources, particularly because publication bias is explicitly planned for assessment. The restrictions to English-language studies and publications from January 2008 onwards should be explicitly justified, as both restrictions could introduce selection bias. Ideally, the final strategy should be reviewed by an information specialist or using a structured peer-review approach such as PRESS.

Response: We agree that the original search was overly restrictive. We have expanded the strategy to include additional trial and intervention terms and specific screening procedures and suspected-cancer referral pathways. The review is not restricted to digital/mhealth interventions and therefore we did not feel it was appropriate for these terms to be restrictive in the primary search strategy. Instead, details of the nature of the intervention (if digital/mHealth) will be extracted once studies are identified as being included. This is specified in the methods. We have added searches of ClinicalTrials.gov and the WHO International Clinical Trials Registry Platform for ongoing and unpublished trials, alongside reference-list searching and updated searches before final analysis. We now explain the January 2008 restriction as a focus on contemporary pathways and interventions, acknowledge the potential selection bias from this date restriction, and explain that the English-language restriction reflects limited translation resources. The protocol also states that information-specialist review using PRESS will be sought where feasible.

Methods, Lines 181-204: *"**Search strategy:** The search strategy will combine terms relating to three key concepts: physical activity and sedentary behaviour; cancer screening or investigation of suspected cancer; and trials or interventions designed to promote physical activity and/or reduce sedentary behaviour. The MEDLINE strategy will use controlled vocabulary and free-text terms, with trunctation and proximity operators where appropriate. Cancer-related terminology will include general screening and suspected-cancer terms alongside terminology for specific screening procedures and referral pathways. The strategy will be adapted for the syntax and indexing terms of each database. The strategy will be reviewed by an experienced information specialist using the PRESS framework, where feasible, before the final search is undertaken. The search will be repeated prior to final analysis.*

Text 1: Example Medline Search

((intervention or program* or programme* or advice or counsel* or promot* or behaviour change or lifestyle or random* or trial* or controlled trial* or cluster*))*

AND

((inactiv or activ* or walk* or strength* or step* or physical activit* or sedentary behaviour or MVPA or grip or exercis*).ti,ab. or (Sport[Mesh] or Exercise[Mesh] or "Physical Fitness"[Mesh] or "Sedentary Behaviour"[Mesh] or "Motor Activity"[Mesh]))*

AND

(((cancer or neoplas or carcino* or tumour* or tumor*) adj5 (early detection or screen* or early diagnosis or suspected cancer or suspected malignancy or urgent referral or fast track or two week wait or rapid diagnos*)).ti,ab. OR (mammograph* or breast screen* or bowel screen* or colorectal screen* or cervical screen* or cervical smear* or HPV test* or faecal occult blood or fecal occult blood or FIT test* or colonoscop* or prostate specific antigen or PSA test* or lung screen* or low dose CT).ti,ab. OR "early detection of cancer"[Mesh] OR (neoplasm[Mesh] and "mass screening"[Mesh]))"*

Methods, Lines 176-180: *"ClinicalTrials.gov and the WHO International Clinical Trials Registry Platform will also be searched to identify ongoing or unpublished eligible trials. Reference lists of all included studies and relevant systematic reviews and commentaries will be screened to identify additional eligible studies. The electronic database and trial-registry searches will be rerun before the final analysis to identify newly published, registered or completed studies."*

Methods, Lines 187-189: "*The MEDLINE strategy will be reviewed by an experienced information specialist using the PRESS framework, where feasible, before the final search is undertaken. The search will be repeated prior to final analysis."*

Methods, lines 168-171: *"Studies published before January 2008 will be excluded to focus the review on contemporary cancer-screening and diagnostic pathways and contemporary physical activity interventions, including the period in which device-based and mobile-health approaches became increasingly relevant to intervention delivery and outcome assessment."*

Comment 2. Outcome eligibility and prioritization: Outcome eligibility and prioritisation require further specification. It is currently unclear whether objectively measured physical activity or sedentary behaviour is an eligibility requirement, or whether studies using only self-reported outcomes could be included but would not contribute to the primary outcome. Please state this explicitly. Furthermore, studies may report steps/day, MVPA, total activity, sedentary time and several device-derived metrics at multiple follow-up periods. A prespecified hierarchy for selecting outcomes and time points would reduce the possibility of selective inclusion of results. Physical activity and sedentary time should also be treated clearly as distinct outcomes rather than implicitly combined simply because both are device-derived.

Response: We have clarified that objective measurement is a requirement for study inclusion. Objectively measured physical activity and sedentary time are now explicitly separate primary outcomes. Conceptually distinct measures, including steps/day, MVPA and total activity, will be synthesised separately rather than selected according to statistical significance. The assessment closest to the end of the intervention will be prioritised, with longer-term follow-up synthesised separately where sufficient data are available. Physical activity and sedentary time will be treated clearly as distinct outcomes

Methods, Lines 155-166: "Outcomes*: The primary outcomes will be: (1) change in objectively measured physical activity and (2) change in objectively measured sedentary time. Eligible objective measures of physical activity will include pedometer-measured steps per day, accelerometer- or device-derived moderate-to-vigorous physical activity and other device-derived physical activity metrics. Eligible sedentary behaviour measures will include accelerometer- or device-derived sedentary time. Objective measurement is required for a study to be included in the review; eligible studies reporting only self-reported physical activity or sedentary behaviour outcomes will be excluded. Where a study*

reports multiple objective physical activity metrics, conceptually distinct outcomes (for example, steps/day, MVPA and total physical activity) will be synthesised separately rather than selected on the basis of statistical significance. Where multiple follow-up assessments are reported, the assessment closest to the end of the intervention will be prioritised for the primary analysis; longer-term follow-up assessments will be synthesised separately where sufficient data are available."

Comment 3. Quantitative synthesis: The quantitative synthesis plan needs additional detail. The manuscript specifies random-effects meta-analysis, MD/SMD and preference for change scores, but states that the approach for combining different forms of outcome data "will be specified before analysis". Such decisions should preferably be specified in the protocol itself. Please clarify the random-effects estimator, handling of change versus endpoint scores, missing SDs, direction of scales, and selection of follow-up periods. Since cluster-randomised trials are eligible, the protocol should also describe how clustering will be handled when trial estimates have not already been appropriately adjusted. The expression "OP test" for heterogeneity should also be checked; if Cochran's Q test is intended, this should be corrected.

Response: We have expanded the statistical methods to prespecify random-effects meta-analysis using restricted maximum likelihood (REML) for between-study variance, with Hartung–Knapp confidence-interval adjustments where the number of studies permits. We clarify the use of mean differences or standardised mean differences where appropriate, preferential use of change-from-baseline data and use of endpoint data when change data are unavailable. Missing SDs will be derived from reported statistics using established Cochrane methods where possible, with investigators contacted when needed; studies without usable variance data will not enter the relevant pooled estimate. Effect directions will be harmonised and follow-up selection is prespecified. For cluster trials, appropriately adjusted estimates will be used; otherwise effective sample sizes will be adjusted using a reported or justified external ICC, with sensitivity analyses where feasible. We also explicitly name Cochran's Q (χ^2) test for heterogeneity. The revised protocol does not prescribe a single method for pooling change and endpoint estimates together; these will only be combined when methodologically appropriate.

Methods, lines 255-282: "Quantitative synthesis: *Where at least two studies are sufficiently homogeneous in terms of population, intervention, comparator and outcome measurement, a meta-analysis will be undertaken. Given anticipated clinical and methodological heterogeneity, random-effects models will be used, with between-study variance estimated using restricted maximum likelihood (REML). Where the number of studies permits, Hartung-Knapp adjustments will be used for confidence intervals around pooled effects. For continuous outcomes measured using the same metric, pooled mean differences (MDs) with 95% confidence intervals will be calculated. Where studies assess the same underlying construct using different measurement scales or metrics that can appropriately be combined, standardised mean differences (SMDs) with 95% confidence intervals will be calculated. Physical activity and sedentary behaviour will be synthesised separately.. Where both change-from-baseline and endpoint data are reported, change-from-baseline data will be used preferentially; endpoint data will be used where change data are unavailable. Where standard deviations are not reported, they will be derived from other available statistics using established Cochrane methods where possible; study investigators will be contacted where required. If an SD cannot be obtained or derived, the study will not contribute to the relevant pooled estimate. Effect directions will be harmonised so that pooled estimates have a consistent interpretation. Where multiple follow-up assessments are available, the assessment closest to the end of the intervention will be used for the primary analysis, with longer-term follow-up synthesised separately where sufficient data are available. For cluster-randomised trials, appropriately cluster-adjusted effect estimates and standard errors will be used*

where reported. Where clustering has not been accounted for, the effective sample size will be adjusted using the reported intracluster correlation coefficient (ICC), or an appropriate ICC from a comparable study where justified; assumptions will be reported and explored in sensitivity analysis where possible.

Assessment of heterogeneity: *Clinical and methodological heterogeneity will be assessed by examining differences in study populations, cancer-related clinical context, intervention characteristics, comparators, outcome measurement and follow-up duration. Statistical heterogeneity will be assessed using Cochran's Q (χ^2) test and the I^2 statistic. An I^2 value above 50% will be considered indicative of potentially substantial statistical heterogeneity, although interpretation will take account of the number of studies and the magnitude and direction of effects."*

Comment 4. Intervention components and subgroup analyses: Subgroup/component analyses need clearer operational definitions. The proposal to compare "BCTs and intervention functions utilised in effective vs non-effective interventions" requires a priori definition of what constitutes an "effective" intervention. Dichotomising trials according to statistical significance could be misleading, particularly with small studies and heterogeneous outcomes. If the number of studies is limited, descriptive mapping of BCTs/intervention functions against effect estimates may be more defensible than inferring that individual components are associated with effectiveness. Please specify the intended analytic approach and avoid causal interpretation of component-effect associations.

Response: We agree that categorising interventions as "effective" or "non-effective" on the basis of statistical significance would be misleading. We have removed this dichotomy. Behaviour change techniques and intervention functions will instead be mapped descriptively against the direction and magnitude of effect estimates. Exploratory comparisons may be conducted if sufficient studies are available, but any associations will not be interpreted as evidence of causal effects of individual components.

Methods, lines 283-292: *"**Subgroup analyses**: Where sufficient data are available, prespecified subgroup analyses will examine: i) mHealth versus non-mHealth interventions; ii) interventions targeting physical activity alone versus interventions targeting both physical activity and sedentary behaviour; and iii) cancer screening populations versus populations referred for investigation of suspected cancer. Mhealth will be defined as 'medical and public health practice supported by mobile devices, such as mobile phones, patient monitoring devices, personal digital assistants (PDAs), and other wireless devices'[9] . Intervention functions (using the Behaviour Change Wheel[9]) and BCTs (using the Behaviour Change Technique Taxonomy version 1 (BCTTv1)[10]) will be mapped descriptively against the direction and magnitude of effect estimates. Any exploratory component-effect comparisons will be interpreted cautiously and will not be considered evidence of causal effects of individual intervention components."*

Comment 5. Risk of bias: Risk-of-bias assessment should distinguish individually randomised and cluster-randomised trials. RoB 2 provides specific guidance/tools for cluster-randomised trials, and risk-of-bias judgements are generally made for particular results rather than assigning a single generic "study quality" classification. Please specify the relevant RoB 2 approach and align the planned sensitivity analyses accordingly.

Response: We now specify use of the standard Cochrane RoB 2 tool for individually randomised trials and the cluster-randomised trial variant and guidance for cluster trials. Judgements will be made for relevant results/outcomes rather than assigning a generic study-quality rating. Sensitivity analyses will exclude results assessed as having overall high risk of bias, where sufficient evidence is available.

Methods, 239-245: "Risk of bias assessment: *Risk of bias will be assessed independently by two reviewers for each relevant outcome/result. Individually randomised controlled trials will be assessed using the standard Cochrane Risk of Bias 2 (RoB 2) tool[11], while cluster-randomised controlled trials will be assessed using the RoB 2 tool and guidance specific to cluster-randomised_trials. Risk-of-bias judgements will therefore relate to particular results rather than a single generic study-quality classification. Disagreements will be resolved through discussion and, where necessary, consultation with a third reviewer. Risk-of-bias assessments will inform interpretation of the findings and planned sensitivity analyses."*

Comment 6. PRISMA-P, missing information and review status: PRISMA-P reporting and methodological references should be corrected. The manuscript states that the protocol was developed according to PRISMA-P but reference 8 is the 2009 PRISMA Statement rather than the PRISMA-P 2015 statement. Please cite the appropriate PRISMA-P publication and cross-check the submitted PRISMA-P checklist against the manuscript. For example, the checklist indicates complete reporting of data-collection processes, although procedures for contacting investigators or handling missing/uncertain information are not clearly described. The manuscript should also state the current review status and anticipated dates/timeline, as appropriate for a protocol.

Response: We have corrected reference 8 to the PRISMA-P 2015 publication and revised the Methods reporting. The data-extraction procedures now explain that investigators will be contacted when information is missing or unclear and that unavailable data will be handled transparently. The manuscript states that the review is ongoing and anticipates completion within 12 months of protocol publication. The submitted PRISMA-P checklist is now consistent with the revised manuscript.

Methods, Lines 224-228: *"Where information required to determine eligibility, risk of bias or quantitative synthesis is missing or unclear, study investigators will be contacted for clarification or additional data. Where data remain unavailable, analyses will use the information reported in the publication and the implications of missing information will be considered in the risk-of-bias assessment and interpretation of findings."*

Comment 7. Alignment of abstract and Methods: Please align the Abstract and main Methods more closely. The abstract describes adherence as a secondary outcome, whereas the formal secondary objectives focus on intervention functions, BCTs and mHealth versus non-mHealth interventions. Please ensure that primary and secondary outcomes/objectives are defined consistently throughout the abstract, eligibility criteria and synthesis plan.

Response: We have revised the abstract to align with the primary outcomes and formal secondary objectives. Adherence remains an extracted intervention characteristic described in the narrative synthesis rather than a formal secondary outcome. Intervention functions, behaviour change techniques and the comparison of mHealth versus non-mHealth interventions are described consistently.

The abstract methods now reads as follows:*"Methods: This systematic review and meta-analysis protocol has been developed in accordance with Preferred Reporting Items for Systematic Review and Meta-Analysis Protocols (PRISMA-P) guidance. MEDLINE (Ovid), Embase (Ovid), Web of Science Core Collection and the Cochrane Central Register of Controlled Trials will be searched from January 2008 onwards, supplemented by searches of relevant trial registries and reference lists. Eligible studies*

will include randomised and cluster-randomised controlled trials involving adults (≥18 years) who have undergone cancer screening or have been referred for investigation of suspected cancer and subsequently received a non-cancer diagnosis. Interventions must explicitly aim to increase PA and/or reduce sedentary behaviour and be delivered at or following cancer screening, or following referral for suspected cancer and receipt of a non-cancer diagnosis. Primary outcomes will be change in objectively measured PA and change in objectively measured sedentary time. Two reviewers will independently screen studies, extract data and assess risk of bias using the appropriate RoB 2 tool for individually randomised or cluster-randomised trials. Intervention content will be coded using the Behaviour Change Technique Taxonomy. Where appropriate, random-effects meta-analysis will be conducted. Secondary objectives will examine intervention functions and behaviour change techniques in relation to the magnitude and direction of effects and compare mHealth with non-mHealth interventions. Certainty will be assessed using the Grading of Recommendations Assessment, Development and Evaluation approach (GRADE)."

Comment 8. Suggested methodological reference: A recent methodological protocol may strengthen the Methods rationale. The authors could consider referencing Ciaccioni et al., MSCA HOPE - Physical education in hospital schools: A protocol paper for systematic and umbrella reviews with policy framework analysis (Open Research Europe, 2026; doi:10.12688/openreseurope.23993.2). Its relevance is methodological: it provides a recent example in the physical-activity field of prospective, preregistered evidence synthesis combining explicitly defined eligibility criteria and structured appraisal of methodological quality and evidence certainty. The most appropriate location would therefore be in the Methods, immediately after the paragraph describing protocol development/ registration, or when explaining the rationale for prospectively defining appraisal and synthesis procedures. A possible formulation is: "Recent protocols for evidence synthesis in the broader physical activity field have similarly emphasised prospective specification of complementary review methods, methodological appraisal and assessment of evidence certainty (Ciaccioni et al., 2026)."

Response: Thank you for highlighting this recent methodological protocol. We have added Ciaccioni et al. (2026) as an example of prospective specification of eligibility, appraisal and certainty-of-evidence methods in the wider physical activity evidence-synthesis literature.

VERSION 2 - REVIEW

**Reviewer**	**1**
**Name**	**Ciaccioni, Simone**
**Affiliation**	**University of Rome 'Foro Italico' Faculty of Sport and Exercise Sciences, Department of Movement, Human and Health Sciences, Division of Human Movement and Sport Sciences**
**Date**	**16-Oct-2026**
**COI**	

The authors have substantially improved the protocol in response to the previous review (e.g., outcome eligibility clarification, quantitative synthesis, cluster-randomised trials, risk-

of-bias assessment and intervention-component analyses). Nevertheless, several issues remain.

1. The revised search strategies (Supplementary File 1) require further verification to ensure reproducibility. Several syntax inconsistencies remain in the Ovid searches, while the CENTRAL and Web of Science strategies contain the apparent typographical error "investing*" instead of "investigat*". The Web of Science publication-year restrictions also require clearer Boolean grouping. Please validate and correct all database-specific strategies, ideally through PRESS assessment, and provide reproducible searches for the trial registries.

2. References (lines 136–138, 214–227 and 286–289). Several methodological citations remain incorrect or incomplete. Reference 8 still corresponds to the PRISMA 2009 Statement rather than PRISMA-P 2015, despite the response letter indicating that this was corrected. Reference 9 identifies the GRADE Handbook but is also cited for the Behaviour Change Wheel and mHealth. References 10 and 11 are missing from the bibliography. The methodological reference (doi:10.12688/openreseurope.23993.2) reportedly added in response to the previous review is also absent. Please reconcile all citations with the reference list.

3. Statistical analyses (lines 240–277). The revised quantitative synthesis plan is considerably clearer. However, the criteria for applying Hartung–Knapp adjustments and the approach to subgroup comparisons should be specified more precisely. Comparisons between mHealth and non-mHealth interventions should be interpreted as exploratory indirect comparisons unless supported by direct randomised evidence.

4. The review can establish whether interventions delivered within cancer-screening or referral pathways improve physical activity or sedentary behaviour, but cannot independently establish that these pathways represent superior "teachable moments" without appropriate timing comparisons. Please qualify this interpretation. Additionally, specify the current review stage and anticipated completion timeline in the manuscript, as indicated in the response letter, and ensure consistency with the PROSPERO registration and PRISMA-P checklist.

VERSION 2 - AUTHOR RESPONSE

Response to Reviewer 1

Effectiveness of physical activity and sedentary behaviour interventions delivered following cancer screening or suspected cancer referral: a systematic review and meta-analysis protocol

We thank the reviewer for the thoughtful and constructive assessment. Amendments are highlighted in the accompanying marked-up manuscript. Reviewers comments are in blue below, responses are in black and changes are highlighted in italics.

Comment 1. Search strategy and eligibility restrictions: The revised search strategies (Supplementary File 1) require further verification to ensure reproducibility. Several syntax inconsistencies remain in the Ovid searches, while the CENTRAL and Web of Science strategies contain the apparent typographical error "investing*" instead of "investigat*". The Web of Science publication-year restrictions also require clearer Boolean grouping. Please validate and correct all database-specific strategies, ideally through PRESS assessment, and provide reproducible searches for the trial registries.

Response: We thank the reviewer for this constructive comment. We have undertaken a comprehensive review and revision of our search strategies for MEDLINE (via Ovid), Embase (via Ovid), Cochrane CENTRAL and Web of Science Core Collection, informed by the six domains of the PRESS 2015 guideline.

This review considered the translation of the research question into search concepts, Boolean and proximity operators, controlled vocabulary, free-text terminology, spelling and syntax, and the application of limits and filters. We have corrected inconsistencies in the original search strategies and clarified the combination of search concepts across databases. In particular, the Web of Science strategy has been revised to ensure that the publication-year restriction (2008–2026) is explicitly applied to the complete combined search set, using #6 AND PY=(2008-2026). This resolves the ambiguity in the original Boolean grouping.

We have also revised the Cochrane CENTRAL search to ensure that the final Boolean combination correctly incorporates the intervention, physical activity and cancer-screening concepts. The MEDLINE and Embase strategies have been reviewed for consistency of terminology and database-specific syntax.

In response to the reviewer's recommendation regarding trial registries, we have additionally developed search strategies for ClinicalTrials.gov and the WHO International Clinical Trials Registry Platform (ICTRP). These combine physical activity and sedentary behaviour terminology with terms relating to cancer screening, early detection and investigation of suspected cancer. The registry strategies have been adapted to reflect differences in search functionality compared with bibliographic databases. The revised strategies are provided in **Supplementary File 1**, with the aim of improving transparency, sensitivity and reproducibility.

Comment 2. Several methodological citations remain incorrect or incomplete. Reference 8 still corresponds to the PRISMA 2009 Statement rather than PRISMA-P 2015, despite the response letter indicating that this was corrected. Reference 9 identifies the GRADE Handbook but is also cited for the Behaviour Change Wheel and mHealth. References 10 and 11 are missing from the bibliography. The methodological reference (doi:10.12688/openreseurope.23993.2) reportedly added in response to the previous review is also absent. Please reconcile all citations with the reference list.

Response: We thank the reviewer for highlighting the remaining inconsistencies in the methodological references and apologise that these were not fully resolved in the previous revision.

We have reviewed the in-text citations against the reference list and corrected the numbering and attribution of methodological references. The revised manuscript now includes the appropriate PRISMA-P 2015 statement (reference 10), the Behaviour Change Wheel publication (reference 8), and references relating to RoB 2 and GRADE. We have undertaken a final reconciliation of the methodological citations to ensure that each in-text citation corresponds to the appropriate reference in the bibliography.

We thank the reviewer for suggesting this additional methodological reference (doi:10.12688/openreseurope.23993.2). We reconsidered its relevance to the specific methods described in our protocol. However, we did not identify a sufficiently direct methodological application that would justify its inclusion. We have therefore retained the references most directly supporting the methodological approaches employed in this review and have carefully reconciled all in-text citations with the reference list.

Comment 3. The revised quantitative synthesis plan is considerably clearer. However, the criteria for applying Hartung–Knapp adjustments and the approach to subgroup comparisons should be specified more precisely. Comparisons between mHealth and non-mHealth interventions should be interpreted as exploratory indirect comparisons unless supported by direct randomised evidence.

Response: We thank the reviewer for this helpful suggestion. We have further refined the statistical analysis plan to clarify the circumstances in which Hartung–Knapp adjustments will be applied and how subgroup comparisons will be undertaken.

We now specify that Hartung–Knapp adjustments will be used for random-effects meta-analyses involving three or more studies, with appropriate safeguards against implausibly narrow confidence intervals. Where only two studies are available, conventional random-effects confidence intervals will be reported, with explicit acknowledgement of the uncertainty associated with estimating between-study heterogeneity.

We have also clarified that subgroup analyses will be prespecified but exploratory. Formal tests for subgroup differences or random-effects meta-regression will be considered only where the number and distribution of studies permit meaningful comparisons. Otherwise, subgroup findings will be presented descriptively.

Importantly, we have explicitly stated that comparisons between mHealth and non-mHealth interventions will be interpreted as exploratory indirect comparisons unless eligible trials provide direct randomised comparisons. We will avoid drawing conclusions regarding comparative superiority from differences between independently pooled subgroup estimates.

These amendments have been incorporated into the Quantitative synthesis and Subgroup analyses sections.

Methods, lines 248-256: *"Given anticipated clinical and methodological heterogeneity, random-effects meta-analysis will be undertaken where at least two studies are sufficiently comparable. Between-study variance will be estimated using restricted maximum likelihood (REML). For meta-analyses including three or more studies, Hartung–Knapp adjustments will be used to calculate confidence intervals around pooled effects. Where the Hartung–Knapp variance estimate produces a narrower confidence interval than the conventional random-effects method, a modified Hartung–Knapp approach with a variance correction will be applied. The chosen adjustment and any deviations will be documented in the final review.*

Where only two studies are available, conventional random-effects confidence intervals will be presented, and the substantial uncertainty in estimating between-study heterogeneity will be acknowledged. Analyses using alternative confidence interval methods will be considered as sensitivity analyses where appropriate."

Methods, lines 287-297: *"Where at least ten studies contribute to a meta-analysis and sufficient studies are represented within each subgroup, differences between subgroups will be examined using a formal test for subgroup interaction, or random-effects meta-regression where appropriate. The number of studies, distribution of relevant study characteristics and potential confounding will be considered when determining whether formal comparisons are appropriate. Where data are insufficient, subgroup findings will be presented descriptively.*

Comparisons between mHealth and non-mHealth interventions will be regarded as exploratory indirect comparisons between different sets of trials, unless eligible studies directly randomise participants between these intervention types. Differences in pooled effects will not be interpreted as evidence of comparative superiority without considering differences in populations, clinical contexts, intervention intensity, comparators and other potential confounders."

Comment 4. The review can establish whether interventions delivered within cancer-screening or referral pathways improve physical activity or sedentary behaviour, but cannot independently establish that these pathways represent superior "teachable moments" without appropriate timing comparisons. Please qualify this interpretation. Additionally, specify the current review stage and anticipated completion timeline in the manuscript, as indicated in the response letter, and ensure consistency with the PROSPERO registration and PRISMA-P checklist.

Response: We thank the reviewer for this important clarification and agree that the review cannot establish the superiority of cancer-screening or suspected-cancer referral pathways as teachable moments without direct comparisons of intervention timing.

We have revised the Introduction to distinguish between evaluating the effectiveness of interventions delivered within these clinical pathways and determining whether the timing of intervention delivery confers an additional benefit.

The revised manuscript now explicitly acknowledges that, in the absence of direct randomised comparisons between intervention time points or clinical contexts, any conclusions regarding the potential role of these pathways as teachable moments will remain hypothesis-generating.

We have also added a Review status and anticipated completion subsection, describing the stage of the review at the time of protocol revision and the expected timeline for completing study selection, data extraction, synthesis and manuscript preparation.

Introduction, lines 125-132: *"Although cancer screening and investigation of suspected cancer may provide opportunities for behaviour change, this review will not be able to determine whether these clinical pathways represent more effective 'teachable moments' than other points in the healthcare journey unless eligible trials directly compare intervention delivery at different time points or clinical contexts. Rather, the review will establish whether physical activity and sedentary behaviour interventions delivered within these pathways are effective compared with their respective control conditions. Any conclusions regarding the potential importance of intervention timing will therefore be considered hypothesis-generating, unless supported by direct comparative evidence."*

Review status and anticipated completion, line 313-317: "*At the time of protocol revision (October 2026), the review is at the pre-literature search phase. We anticipate completing study selection and data extraction by December 2026, with data synthesis and manuscript preparation completed by April 2027. Any amendments to the registered protocol will be documented and reported in the final review."*
