# Supplementary material for: Effectiveness of physical activity and sedentary behaviour interventions delivered following cancer screening or suspected cancer referral: a systematic review and meta-analysis protocol
Source: BMJ Open. 2026 Oct 29;16(10):e127499. doi: 10.1136/bmjopen-2026-127499 (PMC13723863; doi:10.1136/bmjopen-2026-127499)
Supplement: Supplementary file 2 [file bmjopen-16-10-s001.docx]

**Supplementary Figure: Full search strategies**

**Medline (via Ovid):**

1. (randomised or randomized or trial* or intervention* or program* or advice or promot*).ti,ab,kf.

2. randomized controlled trial/ or controlled clinical trial/ or health promotion/

3. 1 or 2

4. (walk* or strength* or step* or MVPA or exercis* or pedomet* or acceleromet* or fitness or “physical activit*” or inactiv* or sedentary or “sitting time”).ti,ab,kf.

5. exp Exercise/ or exp Sports/ or Physical Fitness/ or Sedentary Behavior/ or Walking/

6. 4 or 5

7. ((cancer* or neoplasm* or malignan* or tumour* or tumor* or carcin*) adj5 (screen* or detect* or diagnos* or investigat* or suspect* or refer*)).ti,ab,kf.

8. Early Detection of Cancer/ or Mass Screening/

9. (mammograph* or colonoscop* or sigmoidoscop* or “faecal immunochemical test*” or “fecal immunochemical test*” or “FIT test*” or “pap smear*” or “cervical smear*” or “HPV screen*” or “two week wait” or “2 week wait”).ti,ab,kf.

10. 7 or 8 or 9

11. 3 and 6 and 10

12. limit 11 to yr="2008 -Current"

**Embase (via Ovid):**

1. (randomised or randomized or trial* or intervention* or program* or advice or promot*).ti,ab,kw.

2. randomized controlled trial/ or controlled clinical trial/ or clinical trial/ or health promotion/

3. 1 or 2

4. (inactiv* or activ* or walk* or strength* or step* or “physical activit*” or MVPA or exercis* or pedomet* or acceleromet* or fitness or sedentary or “sitting time”).ti,ab,kw.

5. exp physical activity/ or exp exercise/ or exp sport/ or physical fitness/ or walking/ or sedentary lifestyle/

6. 4 or 5

7. ((cancer* or neoplasm* or malignan* or tumour* or tumor* or carcin*) adj5 (screen* or detect* or diagnos* or investigat* or suspect* or refer*)).ti,ab,kw.

8. cancer screening/ or mass screening/ or early cancer diagnosis/

9. (mammograph* or colonoscop* or sigmoidoscop* or “faecal immunochemical test*” or “fecal immunochemical test*” or “FIT test*” or “pap smear*” or “cervical smear*” or “HPV screen*” or “two week wait” or “2 week wait”).ti,ab,kw.

10. 7 or 8 or 9

11. 3 and 6 and 10

12. limit 11 to yr="2008 -Current"

**Cochrane CENTRAL (Cochrane Library)**

#1 (inactiv* OR walk* OR strength* OR step* OR "physical activit*" OR MVPA OR exercis* OR pedomet* OR acceleromet* OR fitness OR sedentary OR "sitting time"):ti,ab,kw

#2 MeSH descriptor: [Sports] explode all trees

#3 MeSH descriptor: [Exercise] explode all trees

#4 MeSH descriptor: [Physical Fitness] explode all trees

#5 MeSH descriptor: [Sedentary Behavior] explode all trees

#6 MeSH descriptor: [Walking] explode all trees

#7 #1 OR #2 OR #3 OR #4 OR #5 OR #6

#8 ((cancer* OR neoplas* OR malignan* OR carcino* OR tumour* OR tumor*) NEAR/5 (detect* OR screen* OR diagnos* OR investig* OR suspect* OR refer*)):ti,ab,kw

#9 (mammograph* OR colonoscop* OR sigmoidoscop* OR "faecal immunochemical test*" OR "fecal immunochemical test*" OR "FIT test*" OR "pap smear*" OR "cervical smear*" OR "HPV screen*" OR "two week wait" OR "2 week wait"):ti,ab,kw

#10 MeSH descriptor: [Mass Screening] explode all trees

#11 MeSH descriptor: [Early Detection of Cancer] explode all trees

#12 #8 OR #9 OR #10 OR #11

#13 #7 AND #12

Limit to Cochrane Library publication date from 2008 to 2026

**Web of Science:**

#1 TS=(randomised OR randomized OR trial* OR intervention* OR program* OR programme* OR counsel* OR advice OR promot*)

#2 TS=(inactiv* OR walk* OR strength* OR step* OR "physical activit*" OR MVPA OR exercis* OR pedomet* OR acceleromet* OR fitness OR sedentary OR "sitting time")

#3 TS=((cancer* OR neoplas* OR malignan* OR carcino* OR tumour* OR tumor*) NEAR/5 (detect* OR screen* OR diagnos* OR investig* OR suspect* OR refer*))

#4 TS=(mammograph* OR colonoscop* OR sigmoidoscop* OR "faecal immunochemical test*" OR "fecal immunochemical test*" OR "FIT test*" OR "pap smear*" OR "cervical smear*" OR "HPV screen*" OR "two week wait" OR "2 week wait")

#5 #3 OR #4

#6 #1 AND #2 AND #5

#7 #6 AND PY=(2008-2026)

**ClinicalTrials.gov**

ClinicalTrials.gov will be searched using the Advanced Search interface as follows:

("cancer screening" OR "breast screening" OR "cervical screening" OR "colorectal screening" OR mammography OR mammogram OR colonoscopy OR sigmoidoscopy OR "fecal immunochemical" OR "faecal immunochemical" OR "pap smear" OR "suspected cancer" OR "cancer referral" OR "cancer diagnosis" OR "cancer investigation" OR "early cancer detection" OR "urgent referral" OR "two week wait") AND ("physical activity" OR exercise OR walking OR sedentary OR fitness OR pedometer OR accelerometer OR inactive OR sitting OR step)

**WHO International Clinical Trials Registry Platform (ICTRP)**

The WHO ICTRP Search Portal will be searched using the Advanced Search interface as follows:

("cancer screening" OR "breast screening" OR "cervical screening" OR "colorectal screening" OR mammography OR mammogram OR colonoscopy OR sigmoidoscopy OR "fecal immunochemical" OR "faecal immunochemical" OR "pap smear" OR "suspected cancer" OR "cancer referral" OR "cancer diagnosis" OR "cancer investigation" OR "early cancer detection" OR mammograph* OR colonoscop* OR sigmoidoscop* OR "faecal immunochemical test*" OR "fecal immunochemical test*" OR "FIT test*" OR "pap smear*" OR "cervical smear*" OR "HPV screen*" OR "two week wait" OR "2 week wait")

**AND**

(inactiv* OR walk* OR strength* OR step* OR "physical activit*" OR MVPA OR exercis* OR pedomet* OR acceleromet* OR fitness OR sedentary OR "sitting time")

Recruitment status ALL
